# News and Miscellany

**Published:** 1904-05

**Authors:** 


### News and Miscellany.

DR. W. P. PULLEN, Jacksonville, Texas, died April 10th (ult.) of pneumonia.

DR. H. C. WHITEHEAD, City Physician of Fort Worth, died in that City Aril 22d (ult.) of consumption; aged, 43.

THE *Mississippi Medical Record,* Vicksburg, has been adopted by the State Medical Association of Mississippi as its official organ; and the name has been changed to the *Journal of the State Medical Association of Mississippi.* Always sprightly and interesting, it will "smell as sweet by any other name." We congratulate the Association.

THE great Brazos Valley Medical Society held it seventeenth annual election at Caldwell May 10th and 11th inst. It was a disappointment to the editor that he was not able to be present, as he hoped to be, as the B. V. fellows know how to make it agreeable for visitors.

DR. T. J. BENNETT, of Austin, Councilor for Seventh Medical District, inoculated himself in the left forefinger while removing spiculæ of bone in a suppurating wound at Thorndale May 13th. He has been critically ill, suffering from general septicæmia, and two operations have been performed for abscesses of the hand. At this hour, May 17th, 9 a. m., his condition is favorable to recovery, but still dangerous.

DEATH OF DR. JNO. O. SCOTT, OF SHERMAN.—One by one the leaves are falling. Soon there will be left on earth none of the brave spirits of the immortal Medical Department of the Confederate States. Dr. Scott was a Confederate surgeon. He was a Kentuckian, and a great scholar, in the classics as well as in the literature of medicine. Only a short while ago his distinguished brother, ex-Medical Director Preston B. Scott, of the Mississippi Department, died at his home in Louisville, and now we are called on to chronicle the death of this good and great man. Dr. Jno. O. Scott died at his home in Sherman, Texas, April 8th (ult.)

from effects of surgical operation for relief of cancer of the stomach. He was born in 1837 and was 67 years of age. The citizens of Sherman held a most impressive memorial service and many eulogies were pronounced upon the life of this good, useful, and unselfish man, a high type of the American physician and Christian gentleman. Peace to his soul.

---

Good Location Available.—For sale, a fine large residence, barn and stables, separate office near by. Seven acres of fine land. Price, $3000; half cash, balance to suit. Practice averages $2500 to $3000. Collections 98 per cent. Practice goes with property. Pretty village in a beautiful and rich farming section peopled with German and Bohemian settlers; prompt pay. Nearest competition ten miles. Dr. Breezes, care Texas Medical Journal.

---

East Texas Medico-Chirurgical Association.—The next meeting of the East Texas Medico-Chirurgical Association will convene in the "City of Elberta Peaches and Tomatoes," i. e., Jacksonville, at 9 a. m., May 26th and remain in session two days. The Northeast Texas is hereby invited to be represented, as there will be an effort made to consolidate the two as "The N. E. T.," and, as far as I am concerned, I hope to see the consolidation effected. It must also be remembered that the Cherokee County Association also meets here at that time in joint session with the E. T. M.-C. All members are urged to be present.

E. E. Guinn, Secretary.

---

The Association of Medical Officers of the Army and Navy of the Confederacy will meet at Nashville, Tenn., June 14th, 15th and 16th prox. Dr. Jno. R. Gildersleeve, Tazewell, Va., is President.

---

Report of the Last Meeting of the Board of Medical Examiners for the State of Texas.—Dr. M. M. Smith, Secretary of the Board of Medical Examiners, gave out the following report of the meeting held in Austin, April 21, 22 and 23, 1904: Total number of applicants for examination, including all subjects, 97; total number who appeared for midwifery examination, 4; total number who passed successful examination for midwifery, 3; total who failed to pass the examination for midwifery, 1. Those who passed successful examinations for midwifery are as follows:

Mrs. M. E. Brooking, Star, Texas; Mrs. L. Willig, San Antonio, and Mrs. Mary Fillips, Ammonsville. Total number of applicants to practice medicine and surgery, 93; total number who passed successful examinations, 34; total number of conditioned (that is, are entitled to another examination in one branch), 3; total number who failed, 56; total number of lady applicants to practice medicine and surgery, 1, passed; total number of negroes who applied for examination, 8; total number of negroes who passed, 3; total who failed, 5. Dr. Smith believes the very large number of failures is due to the large number of undergraduates who applied for examination. The Board adjourned to meet in Houston, June 15, 1904. Those who passed successfully their examination in medicine and surgery are as follows: Robert Bailey, Coleman; George S. Beaty, Dallas; G. G. Bell, Tyler; O. T. Bundy, Milford; J. E. Crawford, Shelbyville; John Darst, Lillard; S. V. De Pew, Cleburne; Joe V. Dozier, Mt. Vernon; Wm. M. Drake, Marshall; Everett Lee Dye, Plainview; Wm. P. Farrington, Hearne; E. Marion Foster, Manchester, Ohio; Oscar C. Garrett, Washington; H. J. Germany, South Bosque; B. E. Greet, Dallas; J. H. Hicks, Ben Franklin; L. F. Johnson, Buna; W. J. Johnson, Cookville; Stephen Jordan, Center; Nettie Klien, Texarkana; T. B. McAnally, Cumby; C. L. McClellan, Dallas; W. E. McMordie, Gatesville; Oscar McMullen, Rockport; J. Oscar Meharg, Fort Worth; W. L. Michael, Sherman; C. W. Ory, Dallas; W. G. Priester, Houston; James K. Smith, Texarkana, Aleck Spencer, Temple; Phillip M. Sunday, Washington; M. E. Tadlock, Chico; F. D. Teas, Canadian; C. C. York, Fort Worth.

---

Dr. F. D. Thompson, of Fort Worth, has accepted the chair of surgery in the Medical Department of Baylor University at Dallas, vice Dr. S. E. Milliken, resigned.

---

Death of Mrs. Tabor.—The Journal is pained to announce the death of Mrs. Virginia Tabor, wife of State Health Officer Tabor. This most estimable and beloved lady passed away Saturday, April 30th, at 12:30 p. m., after an illness of less than a week. The whole State was shocked at the sad news. Dr. and Mrs. Tabor were recent additions to the social life of Austin, and in the short year or so they have lived with us they had won all hearts. Their beautiful home was but recently finished, and they were comfortably domiciled, with prospect of long and happy life before them. But the death Angel snatched away its ornament, and left desolate hearts within. Mrs. Tabor was Miss Virginia Williams, of Denton. She leaves two little sons, aged 2 and 7 years, respectively. Our heart goes out in tender sympathy to them and the distressed father.

---

TUBERCULAR PATIENTS will be interested to know that on the hills of Kerr county, on the bluffs of beautiful Guadalupe, one thousand feet above sea level, Dr. C. H. Wilkinson, my old-time friend and colleague in Galveston in the terrible times of 1867, has a delightful camp sanitarium: an ideal place for consumptives in early stages to live and get well. Dr. Wilkinson meantime has an office in Hicks building, San Antonio, where he can be reached by letter or consultation in person.

---

DR. M. K. LOTT, so well known in connection with the hyoscine treatment of morphinism, etc., has, in conjunction with Dr. Jno. W. Kenney, established a sanitarium in San Antonio for drug and whisky cases. See their announcement in advertising pages.

---

SAMPLE COPIES.—A few "sample copies" are sent out occasionally. Let it be understood that it is to solicit subscription. They cost money. Should a subscriber receive a "sample copy" (they are addressed from a directory), let him hand it to some one not a subscriber and secure his subscription for me. She is booming. Don't get left, Doctor!

